# Penile alterations at early stage of type 1 diabetes in rats

**DOI:** 10.1590/S1677-5538.IBJU.2016.0454

**Published:** 2017

**Authors:** Mingfang Tao, Cemal Tasdemir, Seda Tasdemir, Ali Shahabi, Guiming Liu

**Affiliations:** 1 Department of Urology, Case Western Reserve University School of Medicine, Cleveland, OH, USA;; 2 Department of Urology, Inonu University, Medical Faculty, Malatya, Turkey;; 3 Department of Pharmacology, Inonu University, Medical Faculty, Malatya, Turkey;; 4 Department of Surgery, MetroHealth Medical Center, Case Western Reserve University, Cleveland, OH, USA

**Keywords:** Diabetes Mellitus, Penis, Erectile Dysfunction

## Abstract

**Objective:**

Diabetes affects the erectile function significantly. However, the penile alterations in the early stage of diabetes in experimental animal models have not been well studied. We examined the changes of the penis and its main erectile components in diabetic rats.

**Materials and methods:**

Male Sprague-Dawley rats were divided into 2 groups: streptozotocin (STZ)-induced diabetics and age-matched controls. Three or nine weeks after diabetes induction, the penis was removed for immunohistochemical staining of smooth muscle and neuronal nitric oxide synthase (nNOS) in midshaft penile tissues. The cross-sectional areas of the whole midshaft penis and the corpora cavernosa were quantified. The smooth muscle in the corpora cavernosa and nNOS in the dorsal nerves were quantified.

**Results:**

The weight, but not the length, of the penis was lower in diabetics. The cross-sectional areas of the total midshaft penis and the corpora cavernosa were lower in diabetic rats compared with controls 9 weeks, but not 3 weeks after diabetes induction. The cross-sectional area of smooth muscle in the corpora cavernosa as percentage of the overall area of the corpora cavernosa was lower in diabetic rats than in controls 9 weeks, but not 3 weeks after diabetes induction. Percentage change of nNOS in dorsal nerves was similar at 3 weeks, and has a decreased trend at 9 weeks in diabetic rats compared with controls.

**Conclusions:**

Diabetes causes temporal alterations in the penis, and the significant changes in STZ rat model begin 3-9 weeks after induction. Further studies on the reversibility of the observed changes are warranted.

## INTRODUCTION

The U.S. Centers for Disease Control and Prevention estimated in 2010 that diabetes affects 25.8 million people in the United States, or 8.3% of the population (1). Erectile dysfunction (ED) is a significant complication for a large number of men with diabetes. Reports of the prevalence of ED in diabetic men range from 27.5% to 75%, depending on age and disease severity (2, 3). In the Massachusetts Male Aging Study sample, the prevalence of ED was three times higher in treated diabetic men than in the age-adjusted general population (28% versus 9.6%) (4). Diabetes and obesity account for more than 8 million ED cases in the US, and the annual cost of diabetes- and obesity-associated ED drug treatment has been estimated at more than $4 billion (5, 6). Moreover, studies have shown that ED tends to be less responsive to treatment in diabetic patients than in non-diabetic individuals (4).

The onset of ED generally occurs at an earlier age in men with diabetes than in men in the general population (4), and its prevalence increases with disease duration, being approximately 15% at age 30 years and rising to 55% at age 60 (7). This suggests that the development and progression of diabetes-associated ED are time dependent. Further studies on temporal structural changes of the penis are needed to guide the development of new treatments.

The normal erection requires integrity of the intracavernous structures, including endothelium, smooth muscle, and nerve terminals. One important erectile mechanism is the release of nitric oxide (NO) from nNOS-immunoreactive nerve terminals or endothelial cells in the corpus cavernosum, leading to increased cyclic guanosine monophosphate and decreased cytosolic calcium concentrations in the smooth muscle cells, causing smooth muscle relaxation and ultimately penile tumescence (8). Previous studies of the corpora cavernosa in streptozotocin (STZ)-diabetic rats have shown significant decreases in the smooth muscle and endothelial cell densities 6 months after induction of diabetes (9), and decreased levels of endothelial nitric oxide synthase (eNOS) and nNOS isoforms 12 weeks after diabetes induction (10). However, the time window from no obvious alterations to the significant morphological changes of the penis and its main erectile components, including the corpora cavernosa, cavernous smooth muscle, and nNOS, during the diabetes progression is not well known. In the present study, we measured the temporal changes of morphology, corpus cavernosal smooth muscle, and dorsal nerve nNOS expression in cross sections of the midshaft penis at early stage of STZ-induced type 1 diabetes in a rat model.

## MATERIALS AND METHODS

### Experimental animals

Male Sprague-Dawley rats (280 to 310g, 10 weeks-old, Harlan) housed in a 12 hours light/dark facility with ad libitum access to food and water were used in this study. The animals were randomly allocated to two groups: diabetics (n=12) and age-matched controls (n=9). Diabetes was induced by a single intraperitoneal injection of STZ (60mg/kg dissolved in 0.1M citrate buffer, pH 4.5), and was confirmed by measurement of blood glucose (>300mg/dL) 72 hours after administration of STZ and at the time of euthanasia. The ACCU-CHEK Advantage blood glucose monitoring system (Roche Diagnostics Corporation, Indianapolis, IN) was used to measure the blood glucose levels. At 3 weeks (n=4 in control group, n=6 in diabetic group) or 9 weeks (n=5 in control group, n=6 in diabetic group) after injection, the rats were euthanized by a single intraperitoneal injection of pentobarbital (200mg/kg). The foreskin and shaft skin were removed. The penis was isolated and excised at the level of the ischial arch. The weight and the stretched length from the tip of the glans penis to the end were measured, and then the tissue was fixed with 10% phosphate buffered formalin solution for immunohistochemical staining. All procedures were approved by the Institutional Animal Care and Use Committee of our University (#08150).

### Immunohistochemistry

After fixation, penile tissues were dehydrated and embedded in paraffin. Sections (5μm) from the middle of the body were used for immunohistochemical staining. In brief, the sections were dewaxed and rehydrated in graded ethanol. After heat-induced epitope retrieval with citrate buffer (Dako, Carpinteria, CA), slides were treated with 0.3% hydrogen peroxide in methanol to quench endogenous peroxidase. The sections were then incubated with blocking buffer for 30 min at room temperature. Primary antibody (rabbit anti-α-smooth muscle actin, Abcam, Cambridge, MA; or purified mouse anti-nNOS, BD Biosciences, San Jose, CA) in 1% BSA was applied overnight at 4ºC. After rinsing 3×5 min., the sections were incubated with secondary antibody (biotinylated anti-rabbit or anti-mouse IgG H+L, Vector, Burlingame, CA) for 2 hours at room temperature. The Avidin-Biotin Complex (ABC) staining method was applied. The sections were then counterstained with hematoxylin. No primary antibody control was used to support the specificity of the immunoreactive staining.

### Image analysis

**Figure 1:**
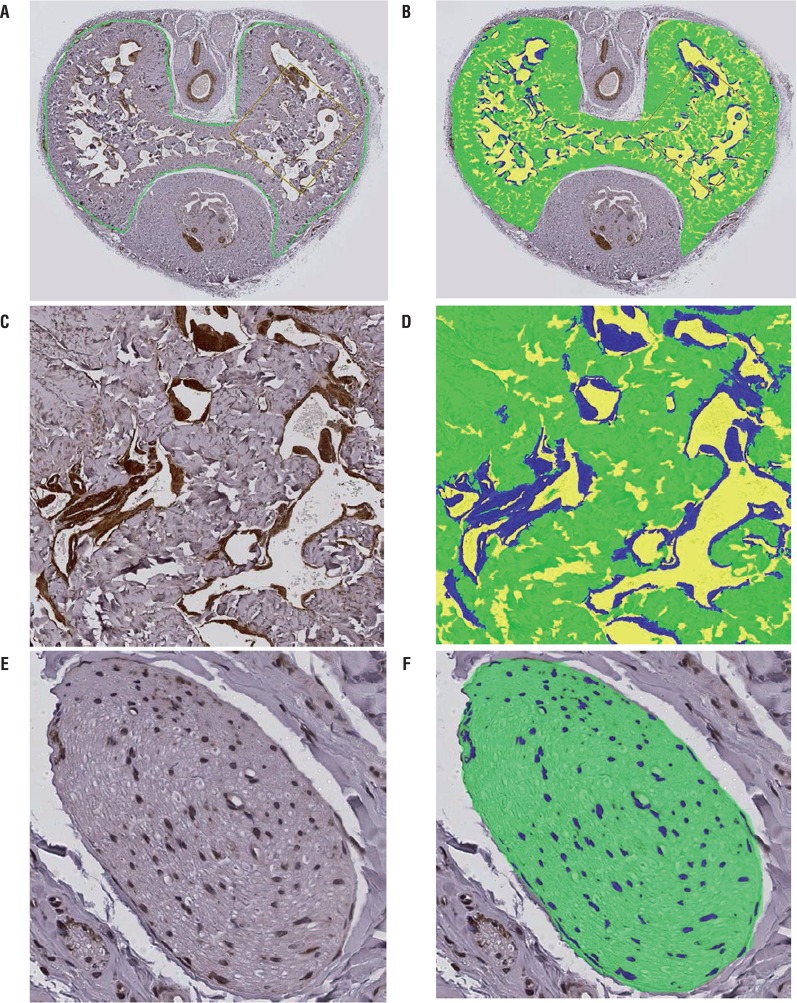
Image analysis method. (A, C) positive immunohistochemical staining of α-smooth muscle actin (dark brown) in a penile midshaft specimen from a control rat. The area of the corpora cavernosa was circled manually in green color in (A). (B, D) software color segmentation performed on the images in (A) and (C) shows the blue-colored smooth muscle and green-colored non-immunoreactive tissue areas that were recognized and captured by the automated digital image analyzer for area measurements, as well as the yellow-colored blank spaces. (E) Positive immunohistochemical staining of nNOS (dark brown) in the dorsal nerve in a penile midshaft specimen from a control rat. (F) software color segmentation performed on the image in (E) shows the blue-colored nNOS and green-colored non-immunoreactive tissue areas that were recognized and captured by the automated digital image analyzer for area measurements.

The stained slides were scanned (Leica SCN 400 Slide Scanner, Leica Microsystems, Buffalo Grove, IL) and digital images of whole cross sections of penile midshaft were saved for analysis using Visiopharm Image Analysis Software (Agern Alle 3, DK-2970 Hoersholm, Denmark), which can distinguish regions stained with different colors and accurately measure the areas by counting the pixels and converting the number of pixels to number of square micrometers. The immunohistochemically-stained α-smooth muscle actin images were used to measure the whole tissue and corpora cavernosa cross-sectional areas. The analysis method is illustrated in Figure-1. The figure includes representative images of immunohistochemically-stained α-smooth muscle actin (brown color) at low (Figure-1A) and high (Figure-1C) magnifications, and nNOS (brown color) at high magnification (Figure-1E). Figures-1B, , and 1f are composite images based on the recognition of different colors by the software. The blue color in Figures-1B and 1D was produced automatically based on the recognition of deep brown color by the software and was used for measuring the α-smooth muscle actin-immunoreactive tissue area. The blue color in the Figure-1F was used for measuring the nNOS-immunoreactive tissue area. The green color in Figures-1B, 1D, and 1F was produced automatically based on the recognition of gray color by the software, indicating non-immunoreactive tissues. The total cross-sectional area of corpora cavernosa or penile dorsal nerves was calculated by adding the green- and blue-colored areas. The yellow colored areas indicate blank space, devoid of any tissue, and were not included in the calculations of tissue areas. The whole tissue cross-sectional area of the penile midshaft was measured using the same method. The percentages of α-smooth muscle actin immunoreactive area in the corpora cavernosa area and nNOS immunoreactive area in the penile dorsal nerve area were calculated. In every case, the processing of images was performed by the same investigator unaware of treatment group assignments.

## Statistics

The data are presented as the mean±standard error of the mean (SEM) for each group. Statistical analysis was done by two-way analysis of variance with Tukey’s multiple post hoc pair-wise comparisons, using Prism 4 (GraphPad, La Jolla, CA). P values <0.05 were considered to indicate statistical significance.

## RESULTS

### General characteristics

General weight and glycemic characteristics of the animals are shown in Table-1. The initial mean body weights of the rats in the diabetic and age-matched control groups were similar, but the diabetic rats weighed significantly less than the controls at 3 weeks and 9 weeks after induction of diabetes with STZ (p<0.05). The mean blood glucose levels of the diabetic rats at 3 and 9 weeks were 4.5 and 4.2 times higher, respectively, than in the control rats (p<0.001). Penis lengths were similar in the two groups at both time points, but penis weight was significantly lower in diabetic rats compared with controls at both 3 and 9 weeks after diabetes induction.

**Table 1:** General characteristics of diabetic and age-matched control rats.

Time Point	Group	n	Initial Weight (g)	Final Weight (g)	Blood Glucose (mg/dL)	Penis Length (mm)	Penis Weight (mg)
3 weeks	Control	4	293.25±2.34	371.25±5.79	132.25±2.25	21.25±0.20	263.50±2.32
	Diabetic	6	298.67±2.53	251.83±9.92*	591.50±2.16*	19.00±0.26	226.17±4.02*
9 weeks	Control	5	293.50±6.13	454.75±14.31	138.50±6.80	22.60±0.22	292.40±11.60
	Diabetic	6	299.17±3.82	220.83±14.12*	576.50±8.54*	21.00±0.26	212.33±6.85*

Values are expressed as mean plus or minus SEM of 4 to 6 individual rats. *significantly different from corresponding value in control group (p<0.01).

### Morphological analysis

As shown in Figure-1A, the body of the penis comprises a pair of corpora cavernosa that are located dorsolateral to the urethra, a corpus spongiosus that surrounds the urethra, and the dorsal nerves and vessels. The cross-sectional areas of the whole tissue and the corpora cavernosa at the penile midshaft were significantly lower in diabetic rats compared with controls at 9 weeks, but not at 3 weeks, after diabetes induction (Table-2).

**Table 2:** Temporal changes of cross-sectional areas of total midshaft penis, corpora cavernosa, smooth muscle as a percentage of corpora cavernosa area, and nNOS as a percentage of dorsal nerves area in diabetic compared with age-matched control rats.

Time Point	Group	n	Area of total penis (mm^2^)	Area of Corpora Cavernosa (mm^2^)	%SMA (%)	%nNOS (%)
3 weeks	Control	4	5.37±0.26	3.93±0.21	10.6±1.4	3.7±0.6
	Diabetic	6	4.76±0.15	3.50±0.12	8.1±0.8	3.7±0.9
9 weeks	Control	5	5.55±0.26	3.89±0.20	9.8±0.5	3.5±0.6
	Diabetic	6	4.31±0.19*	3.17±0.16*	6.6±0.5*	2.3±0.3

Values are expressed as mean plus or minus SEM of 4 to 6 individual rats. *significantly different from corresponding value in control group (p<0.05).

### Smooth muscle in corpora cavernosa

**Figure 2:**
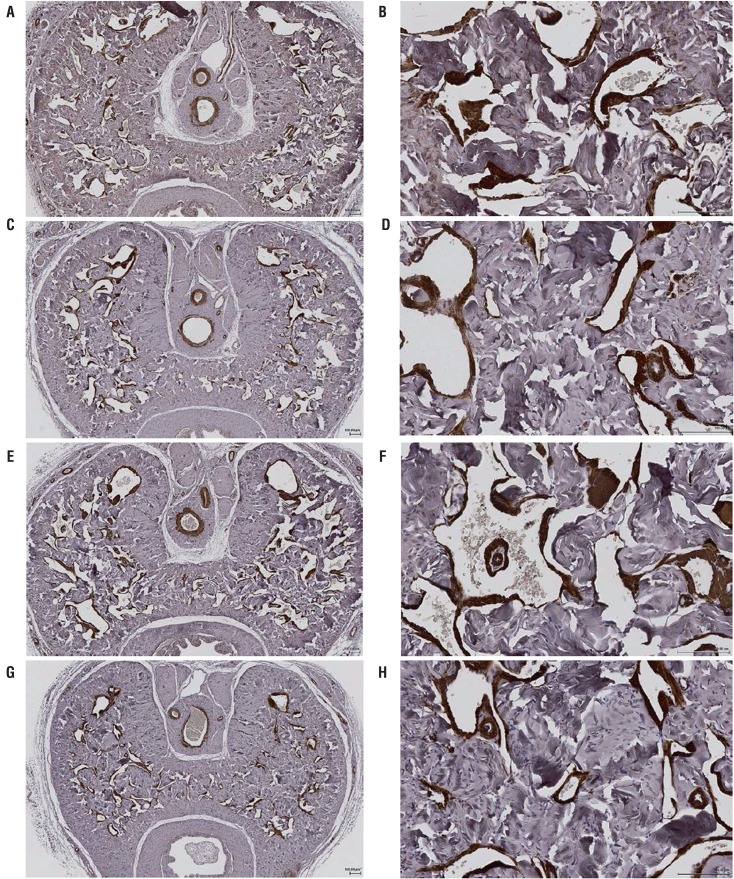
Representative cross-sectional, large field-of-view images of α-smooth muscle actin immunoreactive tissue (dark brown) in penile midshaft sections from 3-weeks (A, B) and 9-weeks (E, F) control rats, and 3-weeks (C, D) and 9-weeks (G, H) diabetic rats, at low (4x) magnification (A, C, E, G) and high (20x) magnification (B, D, F, H).

As shown in Figure-2, smooth muscle cells are important components of corpora cavernosal sinusoids, lying adjacent to the endothelium that lines the sinusoidal space. The sinusoids are separated by dense bundles of connective tissues. There was diffuse positive α-smooth muscle actin immunoreactivity in the controls, whereas diabetic rats exhibited less α-smooth muscle actin immunoreactivity. The cross-sectional area of the smooth muscle within the corpora cavernosa as a percentage of the overall corpora cavernosal area was significantly lower in diabetic rats compared with age-matched control rats at 9 weeks, but not at 3 weeks after induction of diabetes (Table-2).

### nNOS expression in dorsal nerves

**Figure 3:**
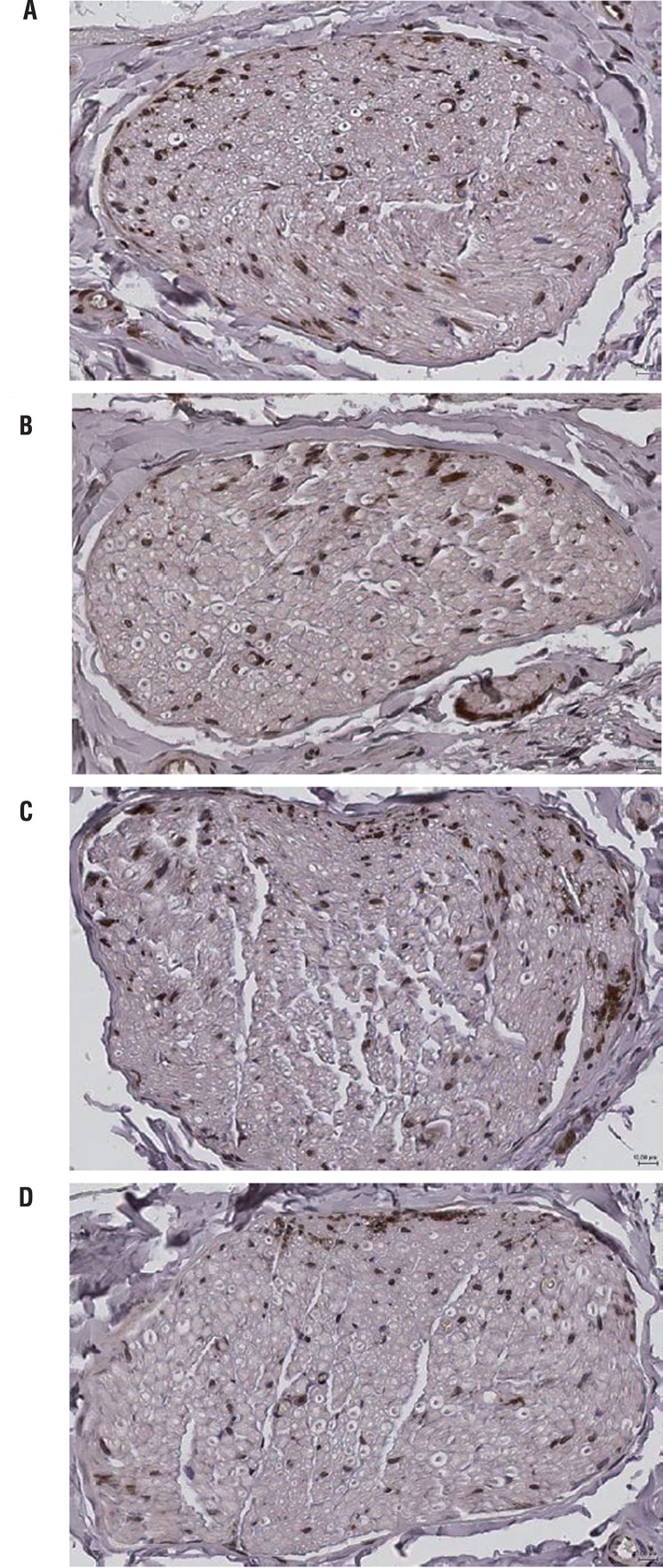
Representative cross-sectional, large field-of-view images of nNOS immunoreactive dorsal nerves (dark brown) in penile midshaft sections from 3-weeks (A) and 9-weeks (C) control rats, and 3-weeks (B) and 9-weeks (D) diabetic rats.

nNOS immunoreactive staining was observed in the dorsal nerves of diabetic and age-matched control rats at both time points (Figure-3). The cross-sectional area of nNOS immunoreactivity as a percentage of dorsal nerve area was similar in diabetic and age-matched control rats at 3 weeks after induction of diabetes, but was decreased at 9 weeks in diabetic rats compared with controls, although the difference was not significant (Table-2).

Magnification 40x.

## DISCUSSION

Studies in experimental animal models and diabetic patients have indicated that the pathogenesis of diabetes-related ED is multifactorial, probably related to central and peripheral neuropathy, endothelial dysfunction, impaired vasodilatory signaling, cavernosal hypercontractility, veno-occlusive dysfunction, hypogonadism, oxidative stress, proinflammatory changes, and psychogenic factors (11, 12). The present study showed that diabetes can cause changes in penile morphology, corpora cavernosal smooth muscle, and dorsal nerve nNOS expression over time.

Our data showed that, while the penis length remained similar between diabetic and control rats, diabetes caused a progressive reduction in total penis weight, from a 14% reduction 3 weeks after STZ injection to a 27.4% reduction at 9 weeks. The reduced weight was due to the decreased main components of the erectile tissue.

The body of the penis from both human being and rat consists of the paired corpora cavernosa, which occupy most of the shaft, are separated by blood vessels and nerves located dorsolaterally, and a corpus spongiosus located ventrally that surrounds the urethra (13). In rats, there is a cylindrical bone that extends from the distal end of the penis body to the tip of the glans. However, it is known that the erection of the penis in mammals, including rats, mainly depends on the engorgement of cavernous spaces with blood and the relaxation of smooth muscle cells (14, 15). Therefore, the corporal smooth musculature plays an important role in the erectile process. Previous studies showed significant smooth muscle loss at 10 weeks (16), 12 weeks (17), 14 weeks (18), or 6 months (9) after diabetes induction. In 7 months old obese Zucker fa/fa rats, a type 2 diabetes mellitus model, Kovanecz et al. (19) found a considerable reduction in penile smooth muscle content. However, the time window from no obvious alterations to the significant morphological changes of the penis is not well investigated. Current study showed that the percentage of smooth muscle in the cross-sectional area of the corpora cavernosa was significantly lower at 9 weeks, but not at 3 weeks, indicating the significant loss of smooth muscle cells happened between 3 to 9 weeks after diabetes induction. The mechanisms of the loss of smooth muscle are not clear. The apoptosis may be one of the pathogenesis. Previous studies have shown significantly increased apoptosis in corporal components from type 1 and type 2 diabetic rat models (20, 21).

Nitric oxide (NO) serves as a principal neurotransmitter in the induction of smooth muscle relaxation (22). NO is generated by the catalytic conversion of L-arginine to L-citrulline by the enzyme nitric oxide synthase (NOS). The primary isoforms of NOS are neuronal (nNOS), endothelial (eNOS), and inducible (iNOS) nNOS and eNOS are constitutively expressed, while iNOS expression is induced by inflammatory stimuli and is associated with immune responses (23, 24). In the penis, eNOS typically is localized in the vascular and sinusoidal endothelium, whereas nNOS is mainly distributed in the non-adrenergic and non-cholinergic nitrergic nerve terminals (23, 24). We stained nNOS in the penile dorsal nerves, rather than in intracorporal tissue, because previous studies have shown that quantification of nNOS in the dorsal nerves is more reliable and is a suitable surrogate for corporal nNOS (25, 26). We found the percentage of nNOS immunoreactivity in the cross-sectional area of the dorsal nerves was similar at 3 weeks and noticeably, but not significantly lower at 9 weeks in diabetic rats, indicating nNOS-enriched nerve fibers were not significant damaged by 9 weeks. However, previous reports have shown reduced nNOS levels in the penis in rats 12 weeks after STZ-induced diabetes (17, 27) and in 26-week old Zucker diabetic fatty rats (26). These results suggested the loss of nNOS and nNOS-enriched nerve fibers could have happened between 9 to 12 weeks. The reduction of nNOS may be caused by a deficiency in antegrade axonal transport of nNOS from the cell bodies, or by accumulation of advanced glycation end products that synergize with NO, leading to oxidative stress and apoptosis of nitrergic nerves (28).

The temporal diabetes-induced changes in the penis inevitably affect the erectile function. Uncovering the time course of these changes is important for the evaluations of drug treatment effects and prognosis. Our study showed that penile weight and morphological measurements, including the important erectile components corporal smooth muscle and nNOS, were not changed significantly at 3 weeks after induction of diabetes. Thus, the first 3 weeks of STZ-induced diabetes is an appropriate time window for observing the preventive effects of new treatments on diabetes-related penile changes. By 9 weeks of diabetes, the penile tissues had changed significantly, at which time the effects of new treatments on the reversibility of diabetes-related ED could be evaluated. Preventative treatments might be expected to have a higher probability of success than later treatments that require reversal of morphological changes. Cho et al. (29) found that phosphodiesterase type 5 inhibitors partially ameliorated ED in 10-week diabetic rats, but were much less effective at 12 and 14 weeks after induction of diabetes. Cellek et al. (30) demonstrated that nitrergic neurons innervating the penis and gastric pylorus lose some of their neuronal nitric oxide synthase content and function less than 8 weeks after diabetes induction, which is followed by nitrergic degeneration more than 12 weeks after diabetes induction. Insulin treatment before 8 weeks reversed the reduction of nitric oxide synthase, but the later nitrergic degeneration was irreversible.

The limitation of this study is that we did not investigate: 1) the erectile function of the rats at the different time points after diabetes induction; 2) if insulin treatment at 3 weeks after diabetes induction can fully prevent the structural changes of penile components. Further studies are needed to answer those questions.

## CONCLUSIONS

Our results show that diabetes affects penile morphology and its components in a temporally progressive manner in STZ-induced diabetic rats, and the alterations are significant at 9 weeks after diabetes induction. Further studies on the reversibility of the observed temporal changes by insulin or new treatments are warranted.
